# Small extracellular vesicles in plasma carry luminal cytokines that remain undetectable by antibody-based assays in cancer patients and healthy donors

**DOI:** 10.1038/s44276-024-00037-x

**Published:** 2024-02-29

**Authors:** Chang Sook Hong, Brenda Diergaarde, Theresa L. Whiteside

**Affiliations:** 1 Department of Pathology, University of Pittsburgh School of Medicine and UPMC Hillman Cancer Center, Pittsburgh, PA 15213 USA; 2 Department of Human Genetics, School of Public Health, University of Pittsburgh and UPMC Hillman Cancer Center, Pittsburgh, PA 15213 USA; 3 Departments of Pathology, Immunology and Otolaryngology, University of Pittsburgh School of Medicine, Pittsburgh, PA 15213 USA

## Abstract

**Background:**

Small (30–150 nm) extracellular vesicles (sEV), also known as exosomes, play a key role in cell-to-cell signaling. They are produced by all cells, circulate freely and are present in all body fluids. Evidence indicates that cytokines are present on the surface and/or in the lumen of sEV. The contribution of intravesicular cytokines to cytokine levels in plasma are unknown.

**Methods:**

sEV were isolated by ultrafiltration/size exclusion chromatography from pre-cleared plasma obtained from patients with head and neck squamous cell carcinoma (HNSCC) and healthy donors (HDs). Multiplex immunoassays were used to measure cytokine levels in paired untreated and detergent-treated (0.5% Triton X-100) plasma and plasma-derived detergent-treated sEV. Non-parametric tests were used to assess differences in cytokine levels.

**Results:**

The presence of cytokines in sEV isolated from patients’ and HDs’ plasma was confirmed by immunoblots and on-bead flow cytometry. sEV-associated cytokines were functional in various in vitro assays. Levels of cytokines in sEV varied among the HNSCC patients and were generally significantly higher than the levels observed in sEV from HDs. Compared to untreated plasma, levels for the majority (40/51) of the evaluated proteins were significantly higher in detergent-treated plasma (*P* < 0.0001–0.03). In addition, levels of 24/51 proteins in sEV, including IL6, TNFRII, IL-17a, IFNa and IFNg, were significantly positively correlated with the difference between levels detected in detergent-treated plasma and untreated plasma.

**Discussion:**

The data indicate that sEV-associated cytokines account for the differences in cytokine levels measured in detergent-treated *versus* untreated plasma. Ab-based assays using untreated plasma detect only soluble cytokines and miss cytokines carried in the lumen of sEV. Permeabilization of sEV with a mild detergent allows for Ab-based detection of sEV-associated and soluble cytokines in plasma. The failure to detect cytokines carried in the sEV lumen leads to inaccurate estimates of cytokine levels in body fluids.

**Figure:**
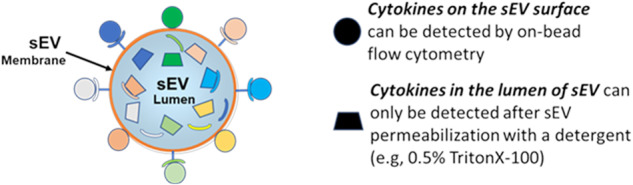
A schema illustrating the presence of cytokines on the surface or in the lumen of small extracellular vesicles (sEV).

## Background

Cytokines and chemokines play a crucial role in the pathogenesis of human diseases [1–3]. Thus, the availability of biologically relevant and reliable methods for their assessment in human body fluids and tissues is critically important. The large number of known cytokines and chemokines and the apparent “division of labor” among them requires reliable methods for defining cytokine profiles and networks. Cytokines work as cascades, and a “cytokine profile” best defines their presence in a constellation of various soluble factors produced by cells. Cytokines mediate cellular interactions by binding to and signaling via cognate receptors on the cell surface [4–6]. Cytokines are local mediators, meant to exert biologic effects in their microenvironment, and systemic effects of cytokines are frequently quite different and more dramatic than those mediated locally: for example, a “cytokine storm” might lead to death [7].

A “cytokine profile” is often dramatically altered in disease (“cytokine polarization”), and measurements of cytokines in body fluids are often used to monitor the presence of inflammatory states or for evaluation of disease progression/regression [8]. Cytokines are proteins or glycoproteins, and antibodies (Abs) specific for cytokines are widely available. Typically, the levels of cytokines in body fluids are measured using Ab-based ELISA or multiplex on-bead assay platforms such as, e.g., Luminex-based assays. The latter has largely replaced the single-cytokine ELISA, providing means for the detection and highly sensitive quantification of numerous analytes in a small volume of body fluids [9, 10].

Emerging data suggest that cytokines are present in body fluids as soluble molecules and also as surface or luminal components of small extracellular vesicles (sEV) otherwise known as exosomes [10]. sEV are 30–150 nm sized vesicles which are formed in multi-vesicular bodies (MVBs) of all cells and are released into extracellular space when MVBs fuse with the cellular plasma membrane [11]. As such, sEV serve as intercellular messengers between cells [12]. They carry proteins indicative of their endocytic origin, such as TSG 101 or ALIX, and tetraspanins (CD9, CD63, CD81) broadly considered as sEV markers [11, 13]. Because the topography of sEV surface proteins resembles that of their parent cells, they are thought to mimic the content of these cells [14].

Tumor cell-derived sEV, often referred to as tumor cell-derived exosomes or TEX, in plasma of cancer patients carry the molecular and genetic content similar to that of tumor cells and, therefore, can potentially serve as a “liquid tumor biopsy.” We and others have reported that TEX isolated from supernatants of tumor cell lines or from body fluids of cancer patients carry cytokines and/or cytokine receptors [15–17]. These proteins are either localized on the surface membrane surrounding each vesicle and/or are found in the vesicular lumen (see Graphical abstract). The vesicular localization of cytokines implies that only surface bound cytokines are accessible to Abs used for their detection in plasma, while luminal cytokines, protected by a protein/lipid bilayer, are not. Mild treatment with a detergent which permeabilizes the sEV membrane without denaturation of the vesicle-associated cytokines would be necessary for detection of such intraluminal analytes using Abs.

Here, we first confirm that cytokines are detectable on the surface and/or in the lumen of sEV and have biological activity in various *in vitro* assays. We then assess levels of cytokines, chemokines and growth factors using Ab-based multiplex immunoassays in paired untreated plasma, mild detergent-treated plasma and mild detergent-treated plasma-derived sEV obtained from patients with head and neck squamous cell carcinoma (HNSCC) and healthy donors (HDs). The objective of the study is to show that cytokines, chemokines and growth factors sequestered in the sEV lumen significantly contribute to their total plasma levels and are unaccounted for when Ab-based assays are conducted using untreated plasma.

## Methods

### Plasma samples from HNSCC patients and healthy donors

Plasma samples were obtained from HNSCC patients seen at the Head and Neck Cancer Clinic of a large healthcare provider under a University of Pittsburgh (Pittsburgh, PA) IRB approved protocol (IRB #: STUDY20030085). Plasma samples from HDs were collected under the same protocol. All study participants provided written informed consent. Blood samples were collected into heparinized tubes and were centrifuged at 1000 x *g* for 10 min. Plasma was stored at −80 ^o^C in 1 mL aliquots. In the initial studies, we used banked plasma samples from 12 HNSCC patients and 3 HDs. For the subsequent studies, we used banked plasma from an independent second cohort including 30 HNSCC patients (stages I to IV) and 10 HDs.

### sEV isolation from plasma

sEV were isolated from plasma, which was pre-cleared by low speed centrifugation, ultrafiltered using 0.22 µm filters and separated by size exclusion chromatography (SEC) using phosphate buffered saline (PBS), as previously described [18]. Vesicles collected in fraction #4 were characterized for size and concentration by NTA (NanoSite NS300, Malvern Pananalytical, Westborough, MA, USA); for vesicular morphology and diameter by transmission electron microscopy (TEM); for protein content of fraction #4 by BCA assays; for the presence of endocytic markers (TSG101, ALIX) in the absence of cytoplasmic proteins, such as Grp94 or calnexin; and the enrichment in tetraspanins (CD9, CD63, CD81) by Western blots. In some cases, when sufficient vesicle numbers were available, their functions were also evaluated, e.g., the ability of TEX to induce apoptosis in activated human T cells, as previously reported [19].

### Membrane-based cytokine Ab array

The initial profiling of cytokines in plasma-derived sEV was performed using a membrane-based cytokine Ab array (Human Cytokine Array C5; Raybiotech, Peachtree Corners, GA, USA) containing 80 individual anti-cytokine Abs positioned on the membrane. Isolated sEV (SEC fraction #4) adjusted to the concentration of 50 µg protein/mL plasma were lysed with RIPA buffer (ThermoFisher, Waltham, MA, USA) containing a cocktail of protease inhibitors (1x concentration, Abcam). The microarray membrane was dipped in the lysate and incubated for 24 h at 4 ^o^C. The arrays were developed and quantified using the Odyssey System (LI-COR imaging systems).

### Luminex-based multiplex immunoassay

We initially used the ProcartaPlex Human Immune Monitoring 65-Plex Panel (Invitrogen, Waltham, MA, USA; Cat # EPX650-10065-901) on the Luminex platform for quantification of cytokines, chemokines and growth factors in untreated plasma and detergent-treated plasma-derived sEV. Plasma samples from 12 HNSCC patients and 3 HDs were utilized. Each plasma sample was divided into two aliquots: one aliquot remained untreated, the other was used for sEV isolation as described above. The isolated sEV (100 µg protein) were subsequently lysed with a mild detergent (0.5% TritonX-100) in a small volume of PBS. The immunoassays were performed as recommended by the manufacturer and evaluated using Bio-Plex Luminex 100 Microplate Reader (BioRad, Hercules, CA, USA). Detection sensitivity of this Luminex-based multiplex assay varies broadly for the different protein targets in the 65-plex panel, with the lower limit of detection (LLOD) ranging from 1 to 407 pg/mL.

### Curiox-based multiplex immunoassay

To increase the detection sensitivity for low abundance protein targets in sEV, we selected to use Curiox “DropArray Bead” plates (Curiox Biosystems, Woburn MA, USA) with the Human Immune Monitoring 65-Plex Panel. The Curiox platform [20, 21] was used to determine cytokine, chemokine and growth factor levels in paired untreated plasma, detergent-treated plasma, and detergent-treated plasma-derived sEV obtained from 30 HNSCC patients and 10 HDs. Each plasma sample was divided into three aliquots: the first aliquot remained untreated, the second was treated with a mild detergent (0.5% TritonX-100), and the third was used for sEV isolation as described above. The isolated sEV from the third aliquot were concentrated to 1ug/uL using ultracentrifugation at 100,000xg for 3 h and were lysed with 0.5% TritonX-100. Assays were performed according to the manufacturer’s protocol. Five uL aliquots of treated/untreated plasma or lysed sEV were placed in each well. All results were normalized to pg protein/mL plasma. Note that for one HNSCC patient, untreated plasma was not available, leaving 29 HNSCC patients with untreated plasma. Detection sensitivity of the Curiox-based assays varies broadly for the different protein targets in the 65-plex panel, with the LLOD ranging from 0.5 to 596 pg/mL.

### sEV flow cytometry for cytokines

For surface staining, sEV were captured on streptavidin beads (ExoCap Streptavidin Kit (MBL International, Woburn, MA, USA) which were coated with a mix of biotinylated Abs specific for tetraspanins (CD9, CD63 and CD81) purchased from Biolegend (SanDiego, CA, USA). For detection of cytokines captured on the surface of beads, labeled mAbs specific for selected cytokines were used as previously described [22]. For detection of total cytokine load in sEV (i.e., cytokines on the surface plus cytokines in the vesicle lumen), isolated sEV were lysed with 0.5% Triton-X100 in PBS, and the solubilized proteins were captured on aldehyde/sulfate-treated Latex beads (4 µm, Invitrogen) before staining with fluorochrome-labeled anti-cytokine detection Abs as previously described [22]. Data are presented as relative fluorescence intensity values (RFV). RFV were calculated as MFI of stained sample/MFI of isotype control.

### Wound healing assay

Aliquots (3 × 10^6^) of SCC-47 cells were plated in wells of a 6-well dish and cultured until cells were 90–95% confluent. Cells were continuously cultured in serum-free media +/− 30ug sEV per well. Prior to addition to the cell culture, sEV were incubated with/without Proteinase K (50ug/mL) for 30 min at 37 ^o^C and then with PMSF (1 mM) for 10 min to stop the Proteinase K reaction. The cell monolayer was wounded mechanically using pipet tips, rinsed with PBS and after 16 h incubation, the wound closure was imaged using Olympus CKX23 inverted microscope at 10X magnification. Data were analyzed using Image J software.

### Transwell migration assays

Aliquots (5 × 10^4^) of SVEC4-10 endothelial cells obtained from ATCC were plated in the upper compartment of 24-well Transwell plates with 8 μm pore diameter membranes (Corning, Durham, NC, USA). Cells were incubated overnight in 100uL of serum free DMEM, 1% (v/v) penicillin/streptomycin (SFM) in the lower chamber with 600uL SFM for overnight starvation. On the following day, the media in lower chambers were supplemented with HNSCC-derived sEV or 10% FBS. Prior to addition to the media, sEV (10 ug protein) were incubated with Abs specific for April, GCSF or IL22 or control IgG (2ug each) for 30 min at RT. After 12 h incubation, non-migrating cells were removed from the upper chamber with cotton swabs. Cell migrated to the lower surface were fixed and stained with 0.2% crystal violets for 30 min and dried. The migrated cells were photographed using Olympus CKX53 microscope at 20x magnification, counted and analyzed using Image J software.

### Statistical analysis

Wilcoxon-Mann-Whitney tests were used to compare continuous variables, e.g., cytokine levels, between HNSCC patients and HDs. Fisher’s exact tests were used to compare categorical variables, e.g., cytokine was detectable yes/no. Wilcoxon signed-rank tests were used to compare cytokine levels between paired samples, e.g., untreated plasma *versus* detergent-treated plasma. Correlation of cytokine level with tumor stage was explored using Spearman correlation, and by comparing cytokine level in HNSCC patients with stage I/II cancer to those with stage III/IV cancer using Wilcoxon-Mann-Whitney tests. *P* values < 0.05 were considered significant. Statistical analyses were performed using SAS^®^ (version 9.4, SAS Institute Inc., Cary, NC, USA).

## Results

### Characteristics of sEV isolated from plasma of HNSCC patients or HDs

**Fig. 1:**
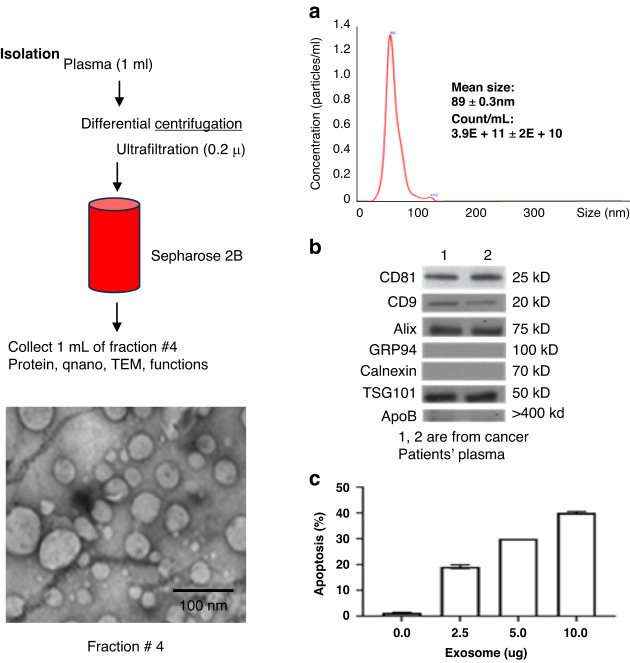
Isolation and characterization of small extracellular vesicles (sEV; also known as exosomes). Plasma was pre-cleared by centrifugation, and sEV were isolated from plasma by ultrafiltration followed by size exclusion chromatography (SEC) on a Sepharose 2B column. sEV were eluted with PBS. Fraction #4 contains non-aggregated vesicles as illustrated by a representative TEM. Fraction #4 is routinely tested for protein, vesicle size by NTA and functional attributes. **a** NTA of sEV in a fraction #4. **b** Western blots of sEV in a fraction #4: note the presence of endocytic markers (ALIX, TSG101) and of tetraspanins (CD9, CD83) and the absence of intra-cellular organelle markers, Grp94 and Calnexin.The sEV carry low levels or no ApoB. **c** sEV in fraction #4 isolated from head and neck squamous cell carcinoma (HNSCC) patients’ plasma induce apoptosis of activated CD8+ T cells in a dose-dependent manner. The presented results are representative for all sEV specimens evaluated in this study.

Methods that are routinely used for isolation and characterization of plasma-derived sEV are summarized in Fig. 1. sEV were isolated by ultrafiltration and size exclusion chromatography (SEC) as previously described [18]. The recovery (measured as protein concentration) of isolated sEV in the SEC fractions #4 ranged from 25 to 140 µg/mL of plasma for the subjects included in this study. The vesicle recovery was higher for plasma from HNSCC patients than for plasma from HDs, as also previously described [23]. The vesicle size ranged from 30–150 nm (mean diameter: 103 nm, standard deviation (sd): ± 14.4), and sEV displayed a vesicular morphology by TEM. Characteristics of sEV isolated from plasma of HNSCC patients or HDs’ plasma were comparable: all vesicles carried one or more tetraspanins (CD9 CD63, CD81), and all were TSG101-positive and ALIX-positive, indicating their endocytic origin, and did not carry cytoplasmic proteins, calnexin or Grp94. ApoB was low or not detectable by western blots in these sEV. According to the currently adopted nomenclature, these vesicles are “small extracellular vesicles” (sEV) [24].

### Detection of cytokines in the lumen and on the surface of sEV

To demonstrate the presence of cytokines in plasma-derived sEV, we initially used a membrane-based cytokine Ab array. The arrays, containing Abs to 80 different cytokines, were overlayed with lysates of sEV isolated from plasma of 12 HNSCC patients and 3 HDs. Semiquantitative results for levels of all 80 cytokines in the 12 HNSCC patients and 3 HDs are reported in STable 1. sEV isolated from HNSCC patients’ plasma contained a greater variety and, generally, higher levels of cytokines than did sEV isolated from plasma of HDs (STable 1). SFig. 1A shows representative results from 3 HNSCC patients and 3 HDs, and SFig. 1B shows results for cytokines that were significantly different between sEV derived from HNSCC patients and those from HDs. Only levels of RANTES (CCL5) were significantly higher in sEV from HDs than in sEV from HNSCC patients.

**Fig. 2:**
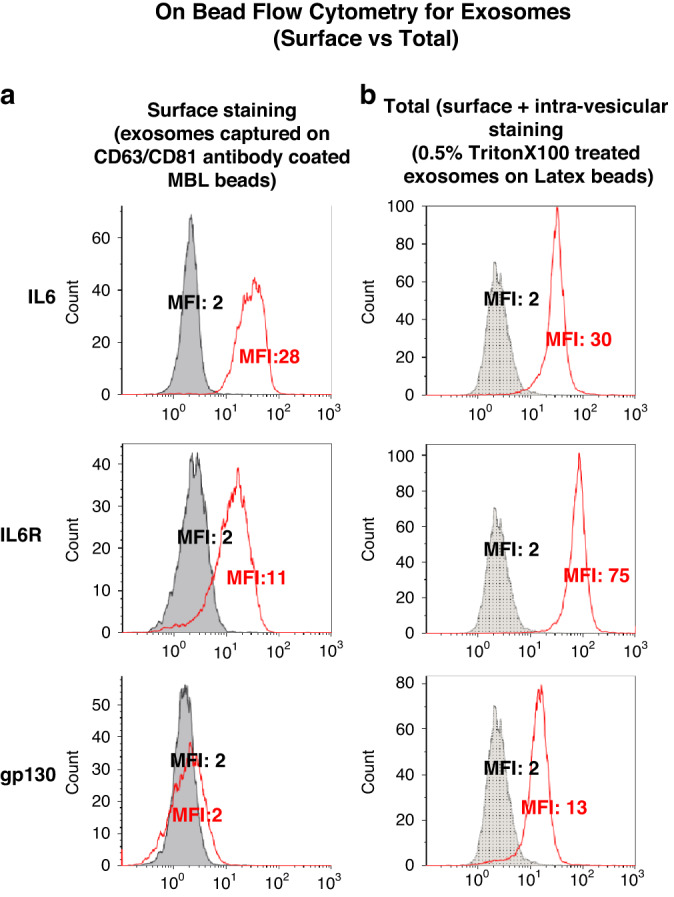
On-bead flow cytometry with sEV (exosomes). Representative results for sEV from a patient with HNSCC. In (**a**), surface staining of sEV captured on anti-tetraspanin Ab-coated beads and detected with fluorochrome-labeled anti-IL6, IL-6R or gp130 antibodies. In (**b**), total (surface plus intravesicular) staining of sEV, which were permeabilized with 0.5% Triton-X100 in PBS and captured on latex beads. Cytokines were detected with fluorochrome-labeled Abs.

On bead-flow cytometry of sEV was performed to interrogate the localization of cytokines on the membrane surface and/or in the vesicular lumen. As previously described by us [22], sEV were captured on streptavidin beads using biotinylated anti-tetraspanin Abs, and the cytokines or cytokine receptors on the membrane surface were detected with selected fluorochrome-labeled detection Abs (Fig. 2). Intraluminal plus surface cytokines/cytokine receptors were detected by flow cytometry following sEV permeabilization with 0.5% TritonX-100 in PBS and capture on latex beads as described in the Methods section, using labeled anti-cytokine detection Abs (Fig. 2b). The data obtained with sEV from plasma of HNSCC patients (*N* = 6) in Table 1 show relative fluorescence intensity values (RFVs) for surface-associated cytokines and for total (surface plus intraluminal) cytokines. Further, the subtraction of RFVs for surface cytokine from RFVs for total cytokine provides a relative measure of intravesicular cytokines. Notably, for 4 of the 9 evaluated cytokines, the relative intravesicular levels were higher than the surface-associated levels (Table 1). These data indicate that a substantial proportion of cytokines associated with sEV reside in the vesicle lumen.

**Table 1:** On bead flow cytometry data for cytokines in plasma-derived sEV from HNSCC patients^a^.

	Mean (± sd) RFV (N = 6 HNSCC patients)	
	Surface	Total
**IL1β**	1.0 ( ± 0.1)	1.3 ( ± 0.6)
**IL6**	2.7 ( ± 1.4)	6.0 ( ± 1.9)
**IL8**	1.6 ( ± 0.5)	7.1 ( ± 3.5)
**IL10**	3.0 ( ± 0.5)	8.8 ( ± 3.8)
**IL17**	2.2 ( ± 1.0)	8.7 ( ± 1.6)
**IFNγ**	1.4 ( ± 0.3)	1.7 ( ± 0.3)
**MCP-1**	3.2 ( ± 0.9)	0.8 ( ± 0.1)
**TNFα**	1.2 ( ± 0.1)	0.9 ( ± 0.1)
**TGFβ**	2.4 ( ± 0.9)	1.4 ( ± 0.6)

^a^sEV were isolated from plasma of six HNSCC patients, and the MFI of each cytokine was determined by on-bead flow cytometry. Relative fluorescence intensity values (RFVs) were calculated as MFI of stained sample/MFI of isotype control.

### sEV carry cytokines which are functional in vitro

To show that cytokines present on the surface membrane of sEV are functional, we compared in vitro results of scratch assays performed with responder cancer cells that were co-incubated with HNSCC plasma-derived sEV. SFigure 2A shows that the addition of sEV to SCC-VII cells significantly promoted cell migration. The treatment of these sEV with Proteinase K, which removes surface proteins, significantly restricted cell migration, suggesting that in sEV, surface-associated cytokines and chemokines may be also involved in modulating the responder cell migration. Although other proteins might modulate EV migration, in our transwell experiments, untreated HNSCC-derived sEV promoted migration of the responder endothelial cells, and neutralizing Abs to April, GCSF or IL-22, but not IgG control Abs, blocked the sEV-driven cell migration (SFigure 2B). sEV treated with 0.5% TritonX-100 were not able to mediate chemotactic activity in either of the two assays.

### Assessment of cytokine levels in plasma and sEV using conventional Luminex-based immunoassays

We initially used the ProcartaPlex Human Immune Monitoring 65-Plex Panel on the Luminex platform to determine levels of cytokines, chemokines and growth factors in paired untreated plasma and detergent-treated plasma-derived sEV from 12 randomly selected HNSCC patients and 3 HDs. Of the 65 target proteins in the panel, only 30 (46.2%) were consistently [≥ lower limit of quantification (LLOQ) in 65% or more of the HNSCC patients or HDs] detected in the untreated plasma (STable 2). In addition, just 12 (18.5%) were consistently detected in the detergent-treated sEV (STable 3). Given the low detection sensitivity, we decided to not move forward with the Luminex platform.

### Cytokine levels in detergent-treated sEV using Curiox-based immunoassays

**Fig. 3:**
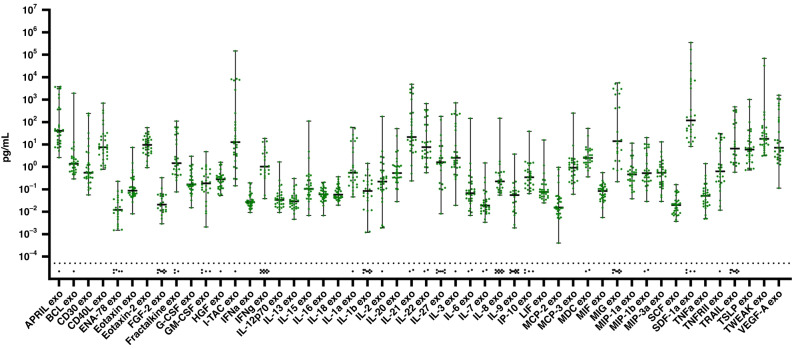
Cytokine levels in detergent-treated sEV using Curiox-based immunoassays. Levels of the 50 proteins detectable in sEV from at least 20 of the 30 HNSCC patients are shown. Each dot represents a subject. Subjects for whom the cytokine level was below the lower limit of detection are shown as black dots below the dotted line. *Y*-axis: log-10 scale. Median with range is shown.

Considering the likelihood that multiple cytokines are sequestered in the sEV lumen but at levels that are too low for conventional detection, we searched for a more sensitive immunoassay capable of detecting small quantities of intraluminal analytes. We selected to use Curiox “DropArray Bead” plates with the Human Immune Monitoring 65-Plex Panel to measure cytokines, chemokines, and growth factors in sEV isolated from plasma of 30 HNSCC patients and 10 HDs. The sEV isolated from plasma were pretreated with 0.5% TritonX-100 for 30 min prior to the assay. Using the Curiox platform, 50 (76.9%) of the 65 target proteins in the panel were detectable in sEV from at least 20 of the 30 HNSCC patients, with 23 proteins detectable in all HNSCC patients (Table 2). As shown in Fig. 3, levels of these 50 proteins in sEV varied broadly among patients. The majority of the 50 proteins (42; 84%) were also consistently detected in the HDs (in 6 or more of the 10 HDs). However, levels in sEV from HNSCC patients were generally significantly higher than levels in sEV from HDs (Table 2). Thus, in HNSCC patients, cytokines enclosed in the sEV lumen appear to represent a considerable albeit variable cargo of proteins that may not be detectable in plasma by immunoassays.

**Table 2:** Cytokine levels in detergent-treated sEV from HNSCC patients and HDs.

Protein	Patients, N (%) ≥ LLOQ (Ntotal = 30)	HDs, N (%) ≥ LLOQ (Ntotal = 10)	Patients, mean (± sd)	HDs, mean (± sd)	*P* value^a^
APRIL	29 (96.7)	9 (90)	633.7 ( ± 1186.52)	15.47 ( ± 24.94)	0.003
BAFF	7 (23.3)	0 (0)	2.2 ( ± 1.4)	ND	
BCL (CXCL13)	29 (96.7)	9 (90)	68.57 ( ± 350.81)	1.55 ( ± 1.48)	0.35
CD30	30 (100)	9 (90)	17.17 ( ± 54.38)	22.63 ( ± 59.42)	0.24
CD40L	30 (100)	9 (90)	56.66 ( ± 139.14)	52.31 ( ± 99.39)	0.34
ENA-78 (CXCL5)	25 (83.3)	7 (70)	0.03 ( ± 0.05)	0.005 ( ± 0.005)	0.02
Eotaxin (CCL11)	30 (100)	10 (100)	0.44 ( ± 1.31)	1.82 ( ± 0.83)	<.0001
Eotaxin-2 (CCL24)	30 (100)	10 (100)	13.7 ( ± 12.29)	28.24 ( ± 12.39)	0.002
Eotaxin-3 (CCL26)	19 (63.3)	5 (50)	0.38 ( ± 0.62)	0.03 ( ± 0.03)	0.02
FGF-2	22 (73.3)	5 (50)	0.04 ( ± 0.07)	0.005 ( ± 0.004)	0.01
Fractalkine (CX3CL1)	27 (90)	9 (90)	15.34 ( ± 25.98)	0.45 ( ± 0.35)	0.002
G-CSF (CSF-3)	30 (100)	10 (100)	0.4 ( ± 0.58)	0.13 ( ± 0.15)	0.02
GM-CSF	26 (86.7)	8 (80)	0.49 ( ± 0.91)	0.51 ( ± 1.09)	0.33
GRO alpha (CXCL1)	14 (46.7)	0 (0)	0.09 ( ± 0.09)	ND	
HGF	29 (96.7)	10 (100)	0.37 ( ± 0.38)	0.22 ( ± 0.16)	0.38
IFNa	30 (100)	10 (100)	0.04 ( ± 0.04)	0.02 ( ± 0.02)	0.052
IFNg	21 (70)	3 (30)	3.73 ( ± 5.11)	0.35 ( ± 0.13)	0.07
IL-10	5 (16.7)	1 (10)	0.12 ( ± 0.13)	0.73 ( ± 0)	0.23
IL-12p70	30 (100)	10 (100)	0.1 ( ± 0.29)	0.03 ( ± 0.03)	0.04
IL-13	30 (100)	10 (100)	0.05 ( ± 0.06)	0.02 ( ± 0.02)	0.2
IL-15	30 (100)	10 (100)	4.03 ( ± 19.65)	2.22 ( ± 5.05)	0.58
IL-16	30 (100)	10 (100)	0.07 ( ± 0.05)	0.04 ( ± 0.02)	0.007
IL-17a (CTLA-8)	17 (56.7)	4 (40)	71.33 ( ± 279.71)	1.71 ( ± 2.44)	0.93
IL-18	30 (100)	10 (100)	0.09 ( ± 0.08)	0.06 ( ± 0.05)	0.13
IL-1a	29 (96.7)	7 (70)	7.61 ( ± 14.6)	3.03 ( ± 7.01)	0.08
IL-1b	22 (73.3)	1 (10)	0.21 ( ± 0.32)	0.05 ( ± 0)	0.71
IL-2	29 (96.7)	9 (90)	6.74 ( ± 32.21)	0.65 ( ± 1.38)	0.52
IL-20	30 (100)	9 (90)	3.4 ( ± 9.83)	2.7 ( ± 5.42)	0.14
IL-21	28 (93.3)	9 (90)	612.78 ( ± 1228.1)	5.47 ( ± 10.33)	0.002
IL-22	28 (93.3)	9 (90)	82.16 ( ± 153.16)	1.98 ( ± 3.5)	0.0004
IL-23	3 (10)	0 (0)	7.29 ( ± 9.6)	ND	
IL-27	23 (76.7)	5 (50)	16.76 ( ± 42.84)	1.7 ( ± 1.18)	0.7
IL-2R	15 (50)	1 (10)	12.1 ( ± 16.77)	0.01 ( ± 0)	0.13
IL-3	29 (96.7)	9 (90)	74.59 ( ± 151.66)	26.31 ( ± 72.27)	0.02
IL-31	11 (36.7)	0 (0)	7.66 ( ± 16.56)	ND	
IL-4	13 (43.3)	0 (0)	1.83 ( ± 2.57)	ND	
IL-5	9 (30)	2 (20)	3.17 ( ± 7.09)	1.24 ( ± 1.15)	0.91
IL-6	28 (93.3)	8 (80)	5.35 ( ± 26.92)	0.12 ( ± 0.2)	0.21
IL-7	28 (93.3)	10 (100)	0.09 ( ± 0.28)	0.08 ( ± 0.18)	0.38
IL-8	21 (70)	3 (30)	7.36 ( ± 31.2)	0.78 ( ± 0.51)	0.6
IL-9	20 (66.7)	0 (0)	0.24 ( ± 0.79)	ND	
IP-10	26 (86.7)	0 (0)	1.98 ( ± 7.28)	ND	
I-TAC (CXCL11)	29 (96.7)	9 (90)	6259.65 ( ± 26683.78)	44.63 ( ± 117.18)	0.055
LIF	30 (100)	10 (100)	0.66 ( ± 2.82)	0.09 ( ± 0.07)	0.46
MCP-1	4 (13.3)	3 (30)	0.06 ( ± 0.05)	0.03 ( ± 0.02)	1
MCP-2	30 (100)	10 (100)	0.09 ( ± 0.22)	0.31 ( ± 0.73)	0.25
MCP-3	30 (100)	9 (90)	9.5 ( ± 44.45)	1.21 ( ± 2.67)	0.04
M-CSF	6 (20)	0 (0)	5.03 ( ± 3.88)	ND	
MDC	28 (93.3)	8 (80)	5.44 ( ± 9.88)	1.04 ( ± 1.01)	0.003
MIF	30 (100)	9 (90)	0.1 ( ± 0.1)	0.27 ( ± 0.19)	0.004
MIG	23 (76.7)	3 (30)	1042.18 ( ± 1678.7)	1.57 ( ± 2.19)	0.03
MIP-1a	30 (100)	10 (100)	1.18 ( ± 2.22)	1.73 ( ± 2.35)	0.41
MIP-1b	28 (93.3)	10 (100)	3.11 ( ± 4.76)	1.7 ( ± 4.61)	0.008
MIP-3a	30 (100)	10 (100)	1.2 ( ± 2.4)	0.36 ( ± 0.4)	0.09
MMP-1	18 (60)	10 (100)	0.14 ( ± 0.12)	0.47 ( ± 0.44)	0.01
NGFb	18 (60)	1 (10)	0.32 ( ± 0.43)	0.02 ( ± 0)	0.17
SCF	30 (100)	10 (100)	0.03 ( ± 0.03)	0.01 ( ± 0.009)	0.07
SDF-1a	26 (86.7)	7 (70)	23586.44 ( ± 73394.65)	327.73 ( ± 765.69)	0.01
TNFa	30 (100)	10 (100)	0.12 ( ± 0.25)	0.05 ( ± 0.07)	0.17
TNFb	7 (23.3)	0 (0)	7.84 ( ± 18.11)	ND	
TNFRII	29 (96.7)	10 (100)	5.76 ( ± 9.17)	3.34 ( ± 9.57)	0.03
TRAIL	22 (73.3)	7 (70)	83.17 ( ± 136.2)	94.9 ( ± 229)	0.12
TSLP	30 (100)	9 (90)	62.81 ( ± 198.44)	58.41 ( ± 147.88)	0.2
TWEAK	30 (100)	10 (100)	3431.98 ( ± 13531.36)	7344.54 ( ± 14747.09)	0.48
VEGF-A	30 (100)	10 (100)	194.21 ( ± 390.15)	111.65 ( ± 328.85)	0.006

^a^Wilcoxon-Mann-Whitney test. *ND* not detected.

### Cytokine levels in paired untreated and detergent-treated plasma using Curiox-based immunoassays

**Fig. 4:**
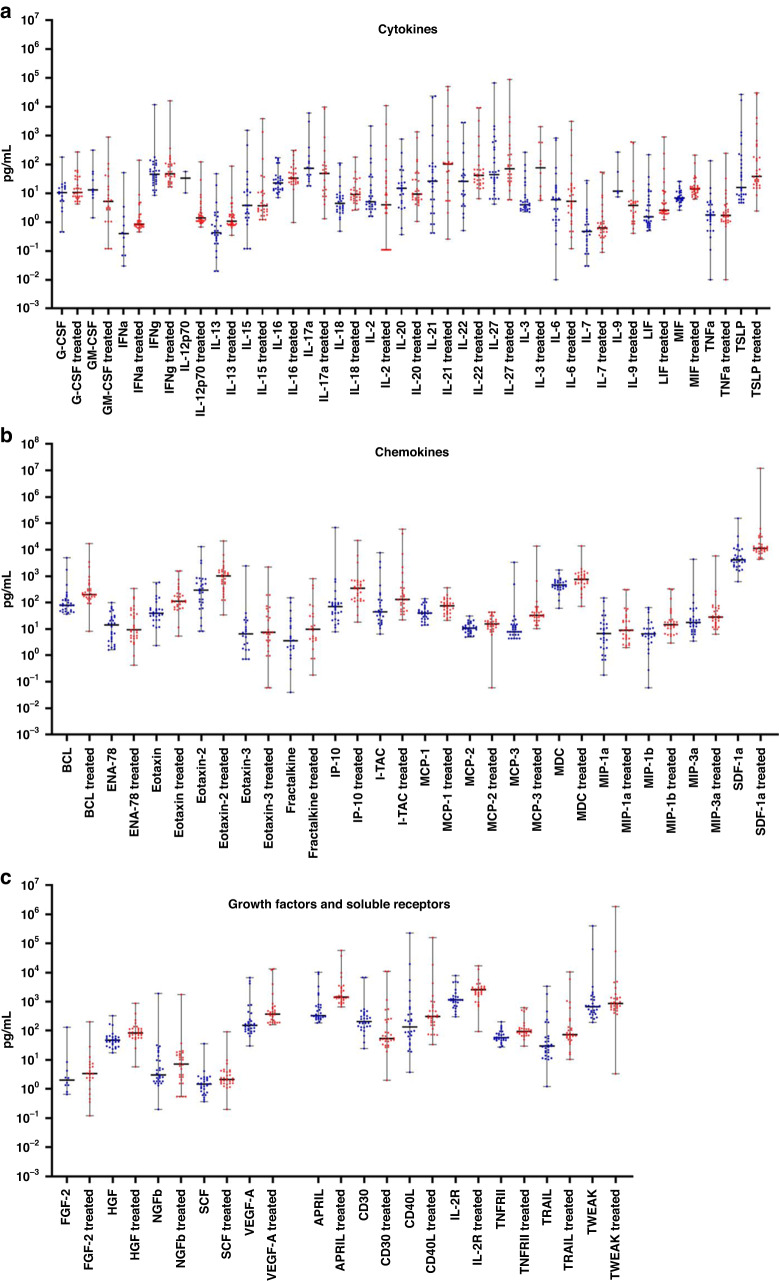
Cytokine levels in paired untreated (blue) and detergent-treated plasma (red) using Curiox-based immunoassays. Levels of the 51 proteins detectable in plasma from at least 20 of the 30 HNSCC patients are shown. Each dot represents a subject. Subjects for whom cytokine level was below lower limit of detection are not shown. Y-axis: log-10 scale. Median with range is shown. Wilcoxon signed-rank test was used to compare levels between detergent-treated and untreated plasma. **a** Shows results for 23 cytokines. All were significantly (*P*: <0.0001–0.03) higher or more commonly detected in detergent-treated plasma except for IL-17a, IL-2, IL-27 and TNFa, which did not differ significantly between untreated and detergent-treated plasma. **b** Shows results for 16 chemokines. All were significantly higher in detergent treated plasma (*P*: <0.0001–0.03). **c** Shows results for 12 growth factors and soluble receptors. All were significantly (*P*: <0.0001–0.002) higher or more commonly detected in detergent-treated plasma except for NGFb (did not differ significantly between untreated and treated plasma), and CD30 (higher in untreated plasma, *P* = 0.03).

We next evaluated cytokine levels in paired untreated and detergent-treated plasma from the same 30 patients with HNSCC using the Curiox platform [20, 21] and the 65-plex panel. Fifty-one (78.5%) of the 65 cytokines, chemokines and growth factors in the panel were detectable in the untreated or the detergent-treated plasma from at least 20 of the 30 HNSCC patients (STable 4). We compared untreated with detergent-treated plasma and found that of the 51 detectable proteins, 40 (78.4%) were present at significantly higher levels in detergent-treated plasma (*P*: <0.0001–0.03) and 4 [7.8%; CD40L, IP-10, Eotaxin-3 (CCL26), and FGF-2] were significantly higher in untreated plasma (*P* <0.0001–0.006) (Fig. 4 and STable 4). Of note, two of the seven proteins for which no significant difference in levels between treated and untreated plasma was observed, IL-9 and IL-12p70, were detected significantly more often in treated than in untreated plasma (STable 4).

**Fig. 5:**
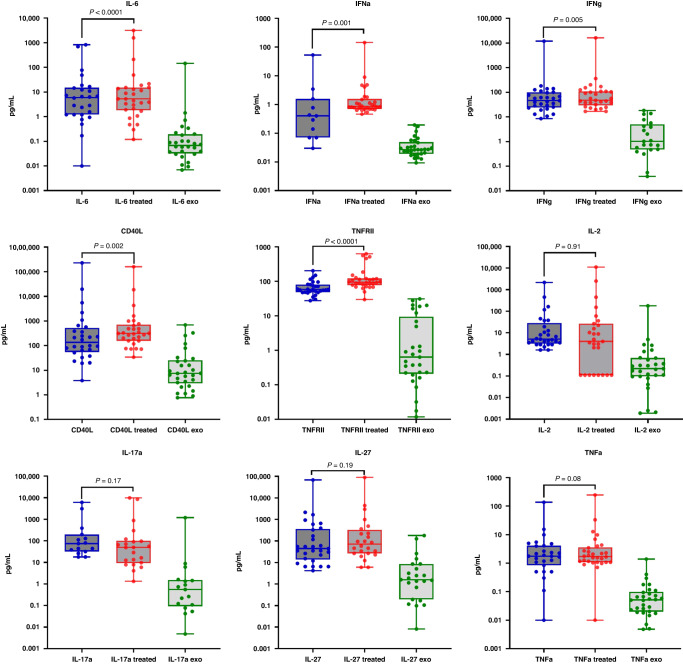
Levels of selected cytokines. Shown are levels in paired untreated plasma (*blue*), detergent-treated plasma (*red*), and detergent-treated plasma-derived sEV (*green*) from HNSCC patients (*N* = 30) using Curiox-based immunoassays. Each dot represents a subject. Boxes show 25th to 75th percentiles, horizontal line is median, whiskers: min to max value. Subjects for whom cytokine level was below lower limit of detection are not shown. *Y*-axis: log-10 scale. Wilcoxon signed-rank test was used to compare levels between paired samples. *P* values are for comparison of untreated plasma with detergent-treated plasma.

Figure 5 shows levels of selected cytokines in paired untreated plasma, detergent-treated plasma, and detergent-treated plasma-derived sEV from the 30 HNSCC patients. Figure 5 summarizes these data for all 51 proteins consistently detected in plasma of HNSCC patients. In Fig. 5, we selected to illustrate several commonly measured cytokines in HNSCC plasma which levels were either significantly different or were not different in untreated vs. treated plasma. In each instance, sEV associated cytokines were present and detectable. Levels in sEV were generally lower than in plasma and varied broadly among patients. In addition, levels in sEV for 24 (47.1%) of the 51 proteins, including IL6, TNFRII, IL-17a, IFNa and IFNg, were significantly positively correlated with the difference between levels detected in detergent-treated plasma and untreated plasma (Table 3).

**Table 3:** Correlation between cytokine levels in sEV and the difference in level between detergent-treated and untreated plasma.

Protein	Target group	rho	*P* value^a^
G-CSF	Cytokines	0.36	0.06
GM-CSF	Cytokines	−0.03	0.87
IFNa	Cytokines	0.44	0.02
IFNg	Cytokines	0.44	0.02
IL-12p70	Cytokines	0.43	0.02
IL-13	Cytokines	0.16	0.40
IL-15	Cytokines	0.94	<.0001
IL-16	Cytokines	0.34	0.07
IL-17a	Cytokines	0.82	<.0001
IL-18	Cytokines	0.15	0.44
IL-2	Cytokines	0.19	0.33
IL-20	Cytokines	0.32	0.09
IL-21	Cytokines	0.79	<.0001
IL-22	Cytokines	0.43	0.02
IL-27	Cytokines	0.03	0.89
IL-3	Cytokines	0.42	0.02
IL-6	Cytokines	0.93	<.0001
IL-7	Cytokines	0.53	0.003
IL-9	Cytokines	0.82	<.0001
IP-10	Cytokines	0.15	0.44
LIF	Cytokines	0.26	0.18
MIF	Cytokines	0.15	0.45
TNFa	Cytokines	0.15	0.45
TSLP	Cytokines	0.05	0.78
BCL	Chemokines	0.20	0.30
ENA-78	Chemokines	0.03	0.89
Eotaxin	Chemokines	0.75	<.0001
Eotaxin-2	Chemokines	0.21	0.27
Eotaxin-3	Chemokines	0.50	0.01
Fractalkine	Chemokines	0.43	0.02
I-TAC	Chemokines	0.60	0.001
MCP-1	Chemokines	0.32	0.09
MCP-2	Chemokines	0.26	0.17
MCP-3	Chemokines	1.00	<.0001
MDC	Chemokines	0.01	0.95
MIP-1a	Chemokines	0.29	0.13
MIP-1b	Chemokines	0.84	<.0001
MIP-3a	Chemokines	0.05	0.81
SDF-1a	Chemokines	0.90	<.0001
FGF-2	Growth factors/regulators	−0.02	0.93
HGF	Growth factors/regulators	0.54	0.003
NGFb	Growth factors/regulators	0.15	0.45
SCF	Growth factors/regulators	0.33	0.08
VEGF-A	Growth factors/regulators	0.82	<.0001
APRIL	Soluble receptors	0.74	<.0001
CD30	Soluble receptors	0.44	0.02
CD40L	Soluble receptors	0.07	0.72
IL-2R	Soluble receptors	0.34	0.08
TNFRII	Soluble receptors	0.72	<.0001
TRAIL	Soluble receptors	0.34	0.07
TWEAK	Soluble receptors	0.40	0.03

^a^Pearson correlation.

### Cytokine levels and disease stage

We next explored whether cytokine levels in detergent-treated plasma or in sEV correlate with disease stage. As shown in STables 5 and 6, levels of a few cytokines were significantly higher in stage II/IV versus stage I/II patients. However, overall we observed no significant correlation with disease stage in this small cohort of patients. We found similar results for untreated plasma (data not shown).

## Discussion

The currently used Ab-based cytokine assays are generally performed using freshly harvested or freshly banked body fluids without adding a detergent. While collection of plasma for cytokine assays requires rapid cooling to prevent proteolytic cleavage of soluble analytes, little attention has been paid to the presence in all body fluids of membrane-bound extracellular vesicles of various sizes and cellular origins which carry intraluminal cytokines. The presence of cytokines on the vesicle surface and in the vesicle lumen has been previously reported [10, 15–17, 25]. Further, studies reported here and elsewhere, consistently show that plasma-derived sEV are functionally competent [26] and modulate functions of immune or non-immune recipient cells via cytokine/chemokine receptor/ligand interactions [27]. These data focus attention on the vesicular cytokines as a critically important component of the cytokine profile in plasma. However, the contribution of vesicle-associated cytokines to measurements routinely performed to evaluate plasma cytokine levels in cancer patients has not been investigated. Here, we show that testing untreated plasma for cytokine levels provides incomplete results, distorting the true cytokine profiles and levels. The vesicles we isolated from plasma and examined for their cytokine content are small extracellular vesicles (sEV) also known as exosomes [24], and they represent only one of many subsets of variously sized vesicles in body fluids. It is likely that larger vesicles, e.g., microvesicles or ectosomes [28], also carry intraluminal cytokines that are not accessible to detection Abs. The treatment with detergent probably releases cytokines in the lumen of small and large extracellular vesicles, providing a crude measure of “hidden” cytokines, chemokines and growth factors packaged into vesicles. The optimal conditions for detergent treatment of vesicles in plasma are yet to be established. It is possible that the detergent by denaturing selected cytokines or by failing to completely release others further distorts the profiles of soluble cytokines measured in Ab-based multiplex assays. Nevertheless, our data, even if not completely accurate because we focused on sEV, serve to raise the awareness of the community that the vesicular compartment present in all body fluids is a silent contributor of cytokines which are being shuttled to cells in a biologically active form. It is important to point out that various other groups of proteins, including biologically active adenosine, for example, are present in sEV lumen and are effectively delivered to recipient cells modulating their functions [29].

The vesicular content of plasma appears to be higher in patients with cancer relative to HDs and varies broadly among different patients, presumably reflecting the rates of vesicle packaging and release by cells. Alternatively, it may be the result of increased level of circulating sEV in patients with cancer relative to HDs [30]. By treating plasma with a mild detergent and performing multiplex detection assays in the presence of detergent, it is possible to release intraluminal analytes into plasma adding them to soluble cytokines and thus increasing levels of the latter. We reported here that levels of 24/51 cytokines detected in sEV were significantly positively correlated with the difference in levels between detergent-treated plasma and untreated plasma (Table 3). We also showed that sEV treated with detergent released various quantities of cytokines which were not denatured, as they were detectable with Abs and had activity in *in vitro* co-incubation assays. We expect that other, larger vesicles in plasma are similarly induced to release their contents upon treatment with detergent. As a result, detergent-treated plasma contains soluble as well as vesicular cytokines and has significantly higher levels of almost all plasma cytokines than untreated plasma. It remains debatable whether 0.5% Triton X100 treatment is sufficient for release and detection of all intraluminal cytokines from all EVs in plasma, especially since EV numbers in plasma from cancer patients are significantly higher than those in HD’s plasma. Nevertheless, our data call attention to the reality of cytokines, chemokines and growth factors being sequestered in vesicles thus preventing their quantification in untreated plasma.

In HNSCC, cytokine profiling in situ in the tumor tissue and/or in body fluids has been often used to evaluate the inflammatory status of the tumor microenvironment and to identify biomarkers of disease progression [30–32]. Cytokines such IL-6, IL-8, IL-10, IL-1β, TNF-α, VEGF, GM-CSF and FGF have been detected in plasma of HNSCC patients and variously correlated with disease stage, progression or survival [33–35]. A recent report used machine learning for predicting outcome to immunotherapy in non-small-lung cancer (NSCLC) based on circulating cytokine signatures [36]. By and large, however, using cytokine signatures for establishing correlations with disease progression or outcome are not consistent, and results of various meta-analyses [37] attempting to link cytokine levels in body fluids with disease activity in cancer or other diseases fail to arrive at strongly significant correlations. We suggest that to date, investigators studying cytokine levels have been unaware that currently used Ab-based cytokine detection methods measure only soluble analytes and do not provide measures of total plasma cytokines. While proteins located on sEV surfaces are detected, intravesicular cytokines are not. This leads to an incomplete analysis and to underestimates in the reports of the total cytokine content in body fluids. In cancer patients, including HNSCC patients, where sEV levels in plasma are significantly elevated [38–40] relative to sEV levels in HDs, the intravesicular cytokines are likely to account for a significant part of total cytokines in plasma. These intraluminal cytokines when released upon detergent treatment are thus likely to significantly contribute to elevated cytokine levels observed in cancer plasma *versus* HD’s plasma.

By comparing cytokine levels between untreated and detergent-treated plasma we observed that the intraluminal cytokine levels and content varied broadly between cancer patients. This is not unexpected, as packaging of individual cytokines into vesicles by cells represents a personalized and probably highly regulated process. Intraluminal cytokines may be utilized for support of tumor growth by autocrine mechanisms [25] or are transported out for tumor-driven reprogramming of non-malignant cells in the TME [41]. Reports in the literature indicate that plasma levels of cytokines in HNSCC patients with stage III/IV disease are elevated relative to patients with stage I/II disease [38–40]. We explored whether cytokine levels in untreated plasma, detergent-treated plasma, and detergent-treated sEV correlated with disease stage using paired samples collected at diagnosis from 30 HNSCC patients. Except for a few cytokines that were significantly higher in stage III/IV patients, we observed no significant correlation with disease stage in our small cohort. This lack of correlation is not surprising: the small number of patients in our study make the possibility of identifying any significant correlation unlikely. Further, recent data point to cytokine signatures or modular “cores” of co-expressed cytokines as best clinical correlates that replace single cytokine assays [42]. Additional larger studies are needed to determine whether detergent-treated plasma specimens will yield more reliable cytokine data that might correlate better with clinicopathological endpoints.

In conclusion, we showed that cytokines sequestered in the lumen of sEV were responsible for differences in cytokine levels between untreated and detergent-treated plasma in HNSCC patients. The data indicated that only soluble cytokines are measured in the untreated plasma, while intraluminal cytokines are not solubilized and thus are not detectable in conventional Ab-based assays. Hence, in the absence of the detergent pre-treatment, commonly used Ab-based assays provide falsely low measurements of cytokine plasma levels. Based on the presented data, accurate Ab-based assessments of cytokine levels in human body fluids will require the use of plasma in which ubiquitous circulating vesicles are induced to release their intraluminal content increasing levels of detectable soluble cytokines.

## Supplementary information

Supplemental Data FINAL

## Data Availability

Data is online and provided upon reasonable request to the authors.
