# Supplementary material for: Small extracellular vesicles in plasma carry luminal cytokines that remain undetectable by antibody-based assays in cancer patients and healthy donors
Source: BJC Rep. 2024 Feb 29;2:16. doi: 10.1038/s44276-024-00037-x (PMC11210721; doi:10.1038/s44276-024-00037-x)
Supplement: Supplementary file 1 — Supplemental Data FINAL [file 44276_2024_37_MOESM1_ESM.docx]

**Supplemental Figure 1**

**
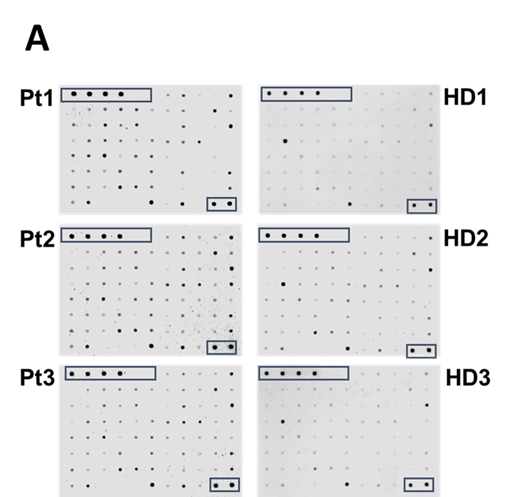
A**

**
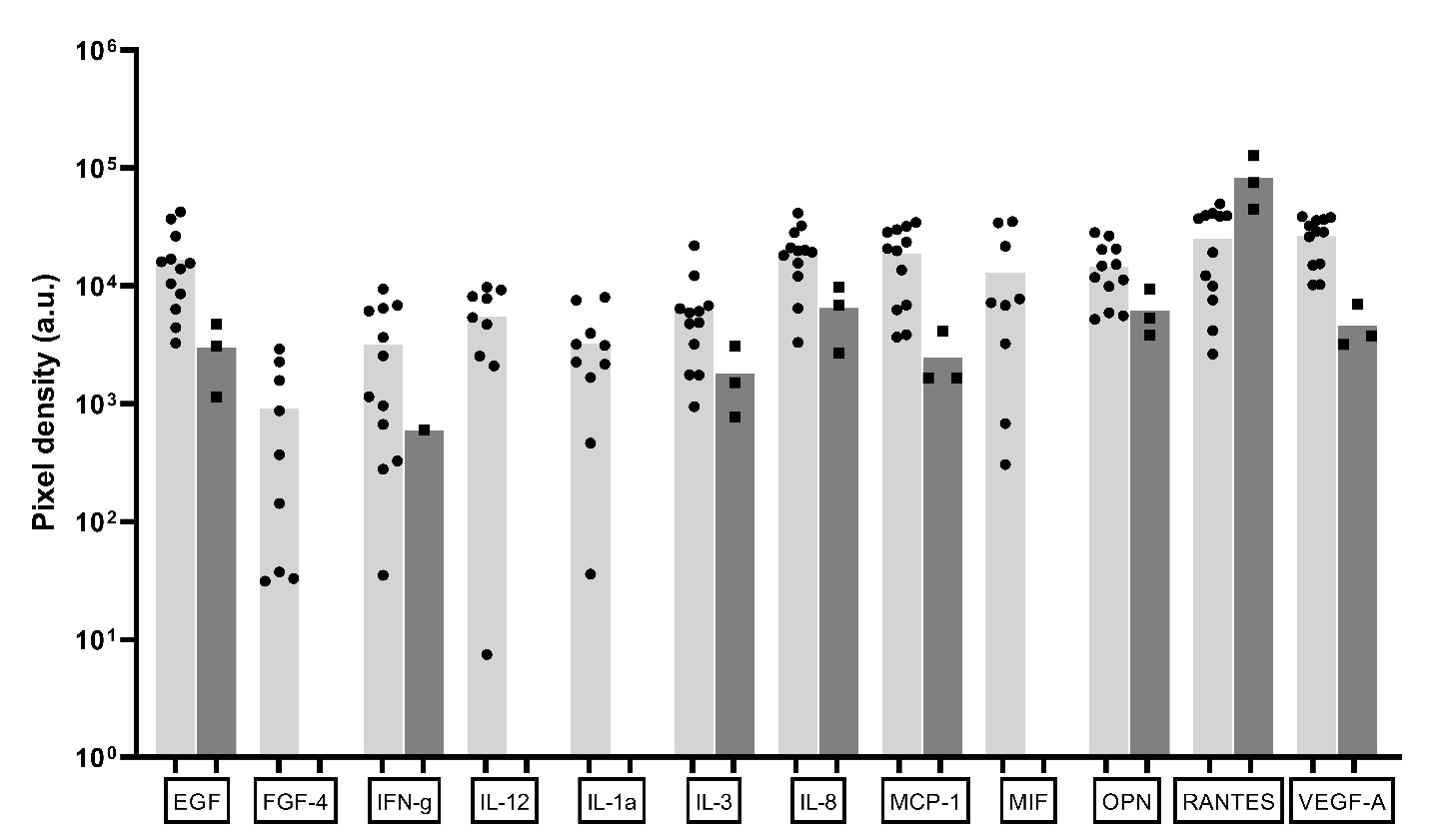
B**

**SFigure 1**: **Membrane-based cytokine antibody arrays with lysates of sEV.** (**A**) Shows representative arrays performed with lysates of sEV isolated from plasma of 3 patients with HNSCC (Pt) and 3 healthy donors (HD). sEV were lysed with 1x RIPA buffer. Membranes were incubated with 50ug protein/mL for 24h. Marked boxes are positive and negative references. (**B**) Semiquantitative results of the antibody arrays for cytokine levels in lysates of plasma-derived sEV from HNSCC patients (N=12) and healthy donors (HDs; N=3). Cytokines that had significantly different levels between sEV from HNSCC patients (light colored bar) and HDs (dark colored bar) are shown. Note: for IL-3, IL-8 and OPN the difference was borderline significant, *P*=0.051; other *P* values ranged from 0.01-0.04. Each dot/square represents a subject; HNSCC patients=dots; healthy donors=squares. Subjects for whom cytokine level was below lower limit of detection are not shown. Y-axis: log-10 scale; top of bar depicts the mean level.

**Supplemental Figure 2**

**
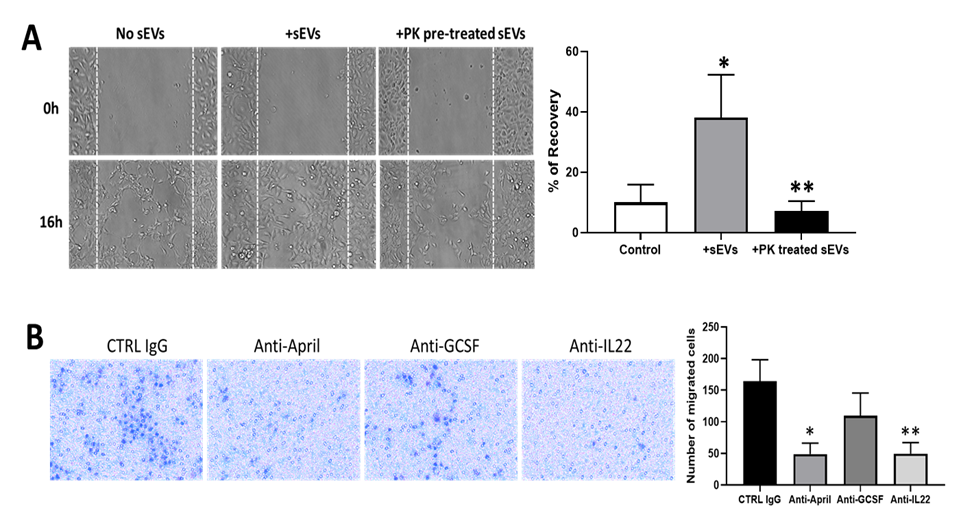
**

**SFigure 2: Functional activity of HNSCC sEV-carried cytokines.** (**A**) Wound healing of SCC-47 cells. After monolayers were scratched mechanically, cells were cultured without supplement (control; No sEVs) or with supplement of 10ug sEV (+sEVs) or with Proteinase K (PK) treated sEV (+PK pre-treated sEVs) for 16hrs. Representative images of migrated cells in 10X magnification are shown. The right panel shows percentage of recovery in the scratched area. Means ± SEM are shown, N=3. *Control *vs.* +sEVs: *P* value <0.02, **Control *vs.* +PK pre-treated sEVs: *P* value >0.2. (**B**) Migration of SVEC4-10 cells towards media containing 10μg of HNSCC sEV treated with antibodies or IgG (2ug) for 30min. Representative images of migrated cells in 20x magnification are shown. The right panel shows number of migrated cells. Means ± SEM are shown, N=4. *CTRL IgG *vs.* anti-April: *P* value: <0.001, CTRL IgG *vs.* anti-GCSF: *P*=0.07, **CTRL IgG *vs.* anti-IL22: *P* value <0.001.

**Supplemental Figure 3**

**
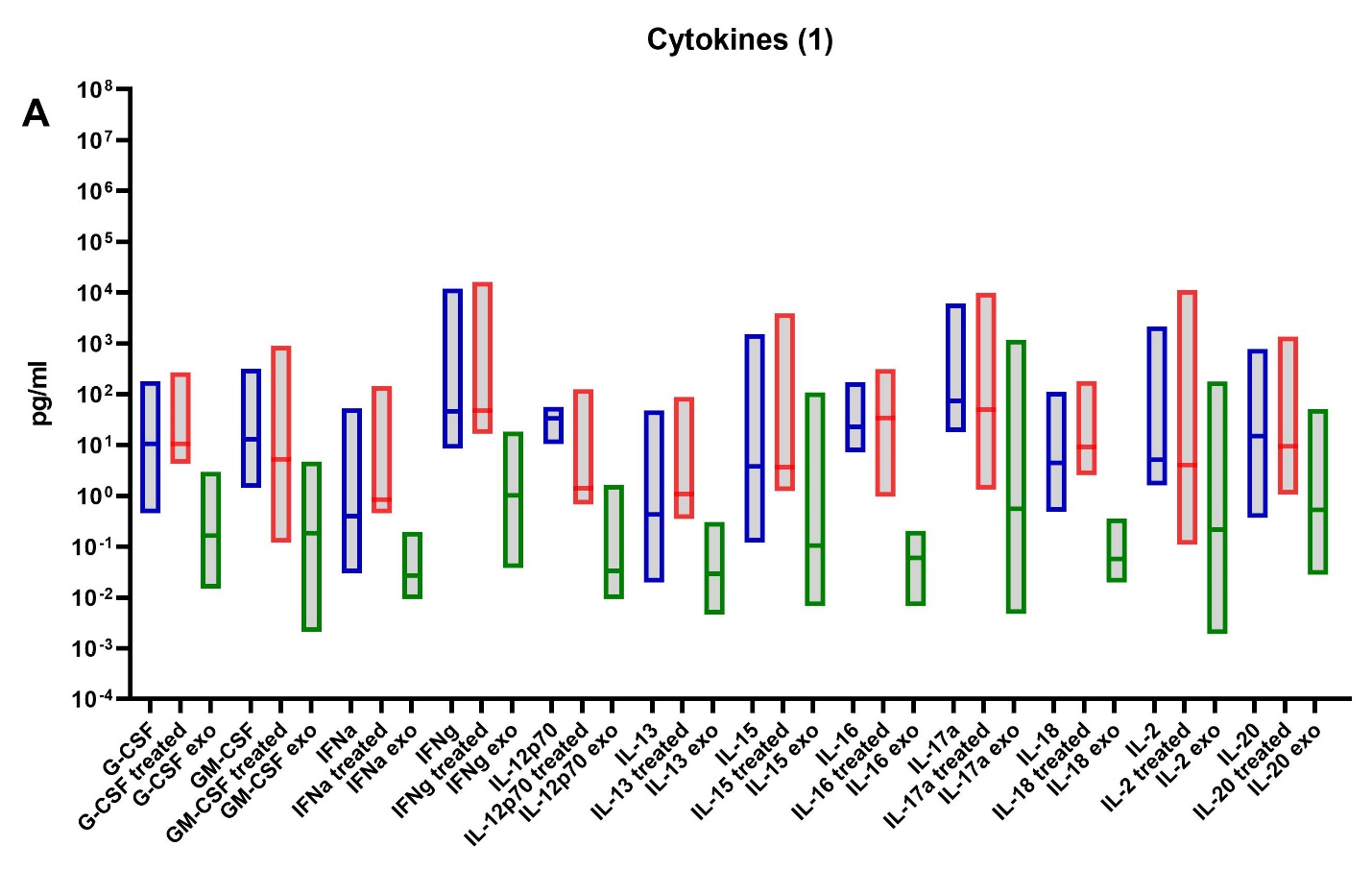
**

**
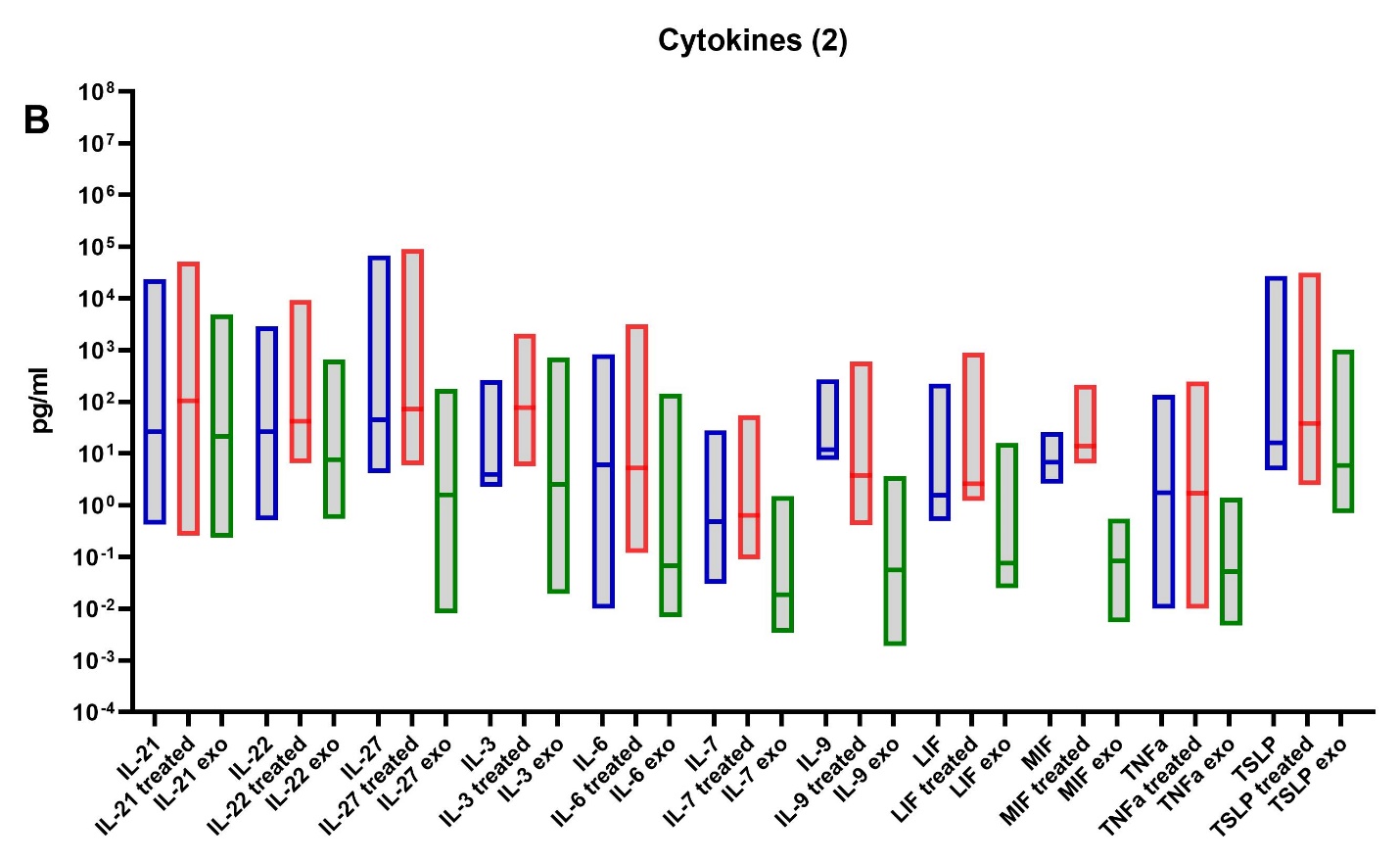
**

**
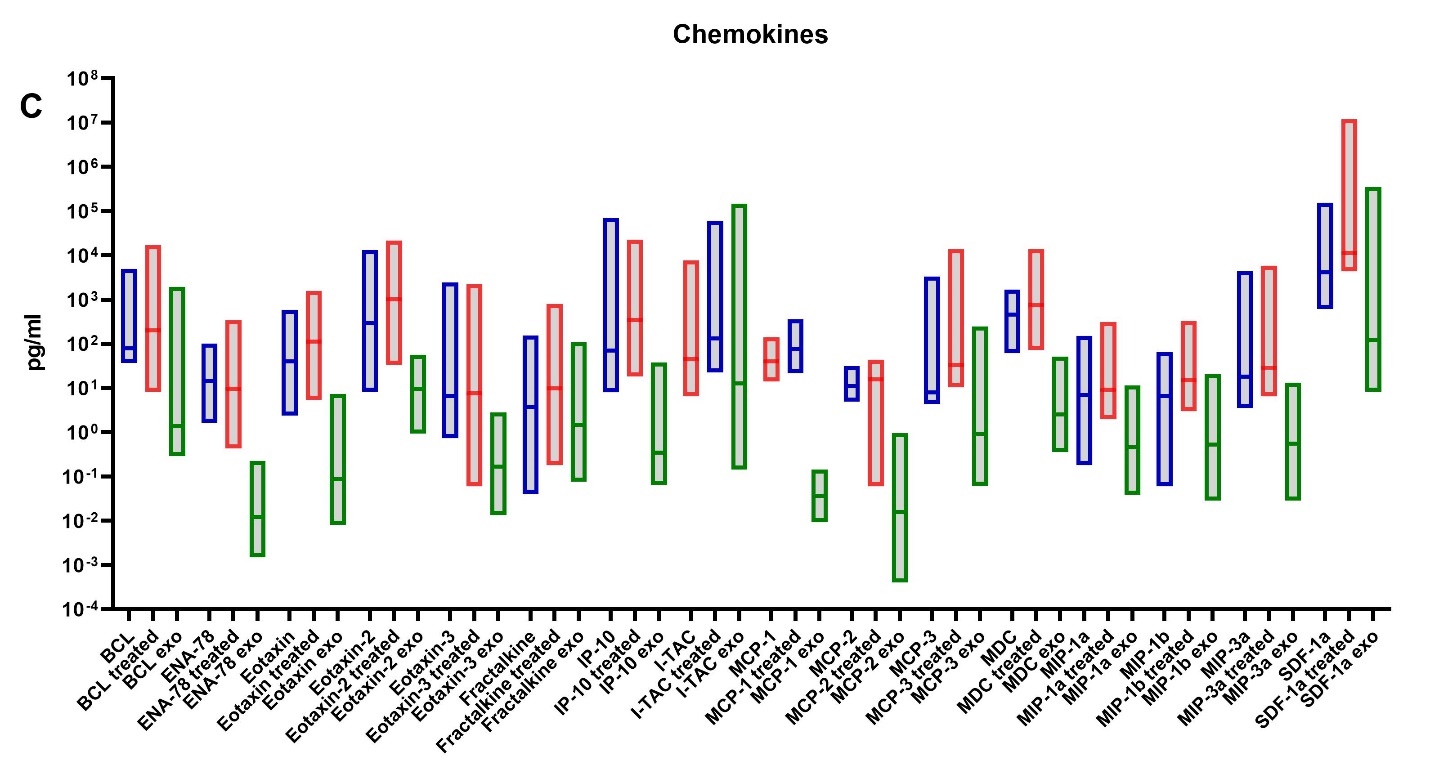
**

**
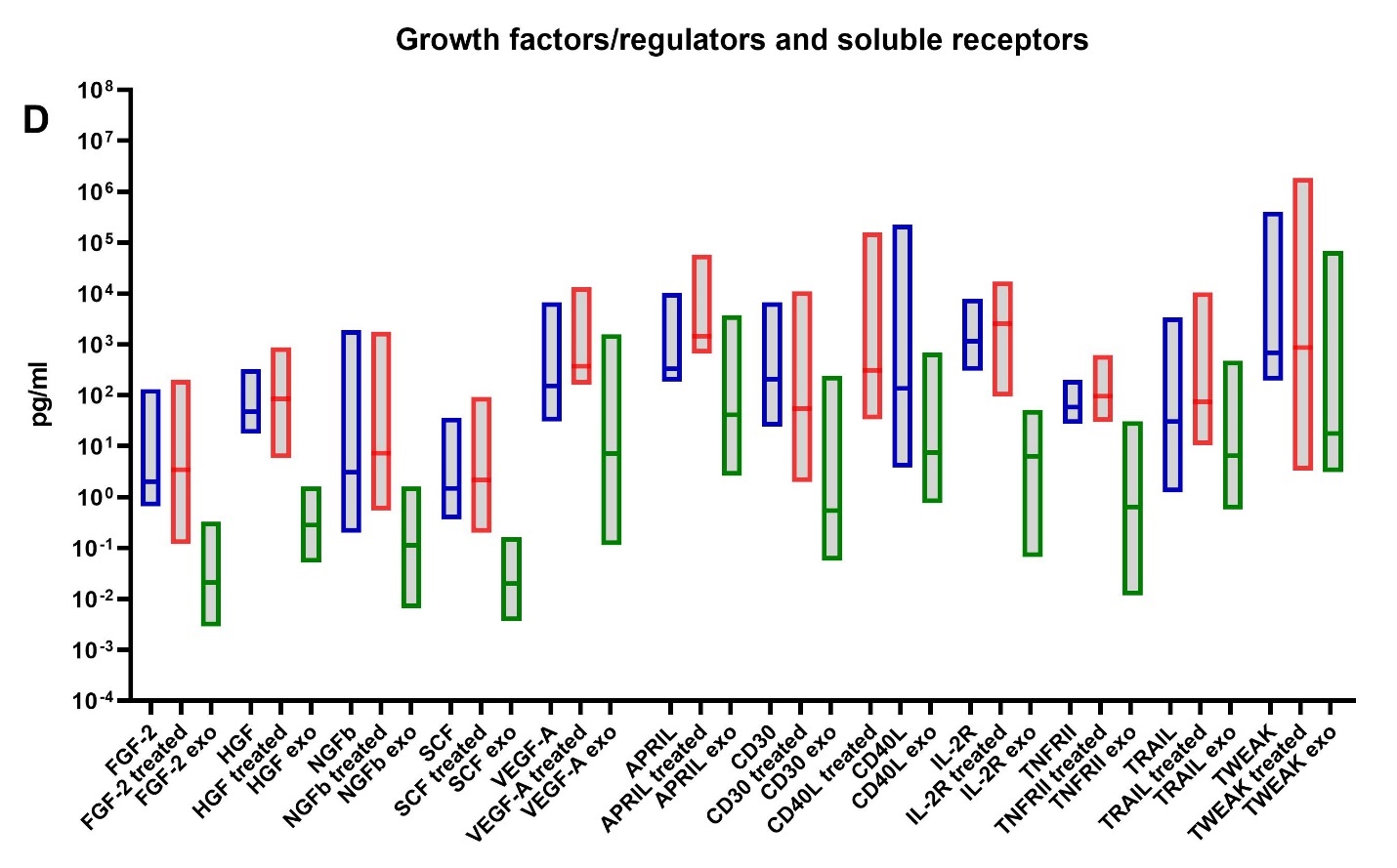
**

**SFigure 3: Cytokine levels in paired untreated plasma (blue), detergent-treated plasma (red), and detergent-treated plasma-derived sEV (green) from HNSCC patients (N=30) using Curiox-based immunoassays.** Levels of the 51 proteins detectable in plasma from at least 20 of the 30 HNSCC patients are shown. Bars show min to max value, horizontal line is median. Y-axis: log-10 scale. Wilcoxon signed-rank test was used to compare levels between the paired samples. (**A**) and (**B**) Show results for 23 cytokines. All cytokines were significantly (*P*: <0.0001-0.03) higher or more commonly detected in detergent-treated plasma than in untreated plasma except for IL-17a, IL-2, IL-27 and TNFa. These four cytokines did not differ significantly between untreated and detergent-treated plasma. IL-3 and IL-21 did not differ between detergent-treated plasma and sEV; all others were significantly (*P*: <0.0001-0.0004) higher in detergent-treated plasma than in sEV. IL-3, IL-9, IL-21 and IL-22 did not differ between untreated plasma and sEV; all others were significantly (*P*: <0.0001-0.008) higher in untreated plasma. (**C**) Shows results for 16 chemokines. All chemokines were significantly (*P*: <0.0001-0.03) higher in detergent-treated plasma than in untreated plasma. All were significantly higher or more commonly detected in detergent-treated plasma than in sEV (*P*: <0.0001-0.003) except for I-TAC (did not differ significantly between detergent-treated plasma and sEV). Except for I-TAC and Fractalkine (did not differ significantly between untreated plasma and sEV), all were also significantly higher or more commonly detected in untreated plasma than in sEV (*P*: <0.0001-0.003). (**D**) Shows results for 12 growth factors/regulators and soluble receptors. All were significantly (*P*: <0.0001-0.002) higher or more commonly detected in treated plasma than in untreated plasma except for NGFb (did not differ significantly between untreated and detergent-treated plasma), and CD30 (higher in untreated plasma; *P*=0.03). All were significantly higher in detergent-treated plasma (*P*: <0.0001 for all) and untreated plasma (*P*: <0.0001-0.01) than in sEV.

**STable 1:** **Results of membrane-based cytokine Ab arrays***

* For the heatmap, pixel density was log10 transformed. White: below lower limit of detection. ND=not detected.

**STable 2: Cytokine levels in untreated plasma from HNSCC patients and HDs using conventional Luminex-based immunoassays**

* Wilcoxon-Mann-Whitney test; ND=not detected.

**STable 3: Cytokine levels in sEV from HNSCC patients and HDs using conventional Luminex-based immunoassays**

* Wilcoxon-Mann-Whitney test; ND=not detected.

**STable 4: Levels in untreated and detergent-treated plasma from HNSCC patients using Curiox-based immunoassays**

***** Wilcoxon signed-rank test

**STable 5: Cytokine levels in detergent-treated plasma and disease stage***

* For the heatmap, cytokine level was log10 transformed. ns: not significant, na: not applicable.

**STable 6: Cytokine levels in sEV and disease stage**** For the heatmap, cytokine level was log10 transformed. ns: not significant.
